# Effects of Extruded Corn with Different Gelatinization Degrees on Feed Preference, Growth Performance, Nutrient Digestibility, and Fecal Microbiota of Weaning Piglets

**DOI:** 10.3390/ani13050922

**Published:** 2023-03-03

**Authors:** Bo Deng, Jie Wu, Xuan Liu, Qian Ma, Xin Tao, Keke Qi, Xinping Diao, Ziwei Xu

**Affiliations:** 1 Institute of Animal Husbandry and Veterinary Science, Zhejiang Academy of Agricultural Sciences, Hangzhou 310021, China; 2 Animal Science and Technology College, Northeast Agricultural University, Harbin 150000, China

**Keywords:** extrusion, corn gelatinization, choice feeding, palatability, growth rate, fecal microbiota, piglets

## Abstract

**Simple Summary:**

This study was conducted to evaluate the effects of extruded corn with different gelatinization degrees on the feed preference, growth performance, nutrient digestibility, and fecal microbiota of weaning piglets. The results showed that extruded corn can improve feed preference, increase growth performance and nutrient digestibility, and modify gut microbiota, and the ideal degree of gelatinization is approximately 41.82–62.60%.

**Abstract:**

Preference and performance trials were conducted to investigate the effects of extruded corn with different degrees of gelatinization on the feed preference, growth performance, nutrient digestibility, and fecal microbiota of weaning piglets. In the preference trial, 144 piglets who were 35 days old were weighed and allotted to six treatments with four replications per treatment. Piglets in each treatment group were allowed to choose two of the following four corn-supplemented diets: conventional corn (NC) or extruded corn with low (LEC; 41.82% gelatinization), medium (MEC; 62.60% gelatinization), or high (HEC; 89.93% gelatinization) degrees of gelatinization for 18 days. The results showed that the piglets preferred diets supplemented with a low degree of gelatinization of extruded corn. In the performance trial, 144 piglets who were 35 days old were weighed and allotted into four treatments with six replications per treatment. Piglets in each treatment were fed one of the four diets for 28 days. The results showed that LEC and MEC decreased the feed:gain ratio at 14–28 days and 0–28 days, respectively, and increased the apparent total tract digestibility (ATTD) of crude protein compared with NC. Meanwhile, LEC increased the total protein and globulin content in the plasma on day 14, and MEC increased the ATTD of ether extract (EE) compared with NC. Extruded corn with low and medium degrees of gelatinization increased the abundance of Bacteroidetes at the phylum level and *Lactobacillus*, *Alloprevotella*, *Prevotellaceae_UCG-03*, and *Prevotella_2* at the genus level. The results showed that extruded corn can improve feed preference, increase growth performance and nutrient digestibility, and modify gut microbiota, and the ideal degree of gelatinization is approximately 41.82–62.60%.

## 1. Introduction

Weaning stress is normally associated with decreased feed intake and growth performance due to abrupt changes in the piglet’s environment and diet [1,2]. It takes up to three weeks for piglets to re-establish their pre-weaning levels of energy intake [3]. Thus, feed with high digestibility and good palatability is crucial to ensure a fast initiation of feeding after weaning and maintain a continuous supply of energy. The nutritional improvement of corn is of interest to producers because corn plays an important role in the piglet diet because it is rich in starch and a major source of energy.

Extrusion is a thermal and mechanical process that results in several starch chemical changes, including changes to the crystalline structure and gelatinization degree that could increase enzyme susceptibility and flavor [4,5,6]. Therefore, corn extrusion may be an effective way to improve nutrition utilization and palatability in the piglet diet.

Many experiments have focused on the effect of gelatinized vs. non-gelatinized corn or its supplementary ratio, yet few results have been published regarding the appropriate gelatinization degree. Different gelatinization degrees of extruded corn may result in different fermentable substrates in the hindgut which change the gut microbiota. Further, starch with different gelatinization degrees would change pellet properties, such as hardness and water absorption indexes, which may affect piglets’ chew work and thus influence palatability [7,8]. Our hypothesis is that the composition of gut microbiota and feed palatability may vary with changes in gelatinization degrees. We noticed that findings in previous studies were inconsistent. Kotara et al., found that the average daily gain (ADG), average daily feed intake (ADFI), and nutrient digestibility increased linearly when the gelatinization degree increased from 23.8% to 81.9% [9]. In contrast, Hongtrakul et al., showed a quadratic decrease in ADG and ADFI and a quadratic increase in the digestibility of dry matter (DM), nitrogen, and gross energy (GE) when the gelatinization degree increased from 14.5% to 89.3% [10]. Therefore, in the present study, we aimed to quantify the palatability of extruded corn with different degrees of gelatinization using a two-way choice–preference trial. The impact of extruded corn with different gelatinization degrees on the growth performance, nutrient digestibility, and fecal microbiota of piglets was also studied in a performance trial.

## 2. Material and Methods

### 2.1. Extruded Corn

All experimental materials of corn from northeast China were selected from a single patch. Corn was grounded using a hammer mill with a 2.0 mm screen and then extruded using a single-screw extruder (EXT-200S, Beijing Modern Yanggong Machinery S&T Development Co., Ltd., Beijing, China). To obtain low, medium, and high gelatinization degrees of the extruded corn, the samples were steam-cooked at 105 °C for 60 s, 80 s, and 100 s with 17%, 17.5%, and 17.5% moisture content and then extruded at 100 °C, 110 °C, and 120 °C for 5 s, respectively. After this, the corn samples were cooled using a counter-flow cooling procedure and ground though a 1 mm screen to generate mashed corn.

### 2.2. Experiment 1: Preference Trial

#### 2.2.1. Experimental Animals, Design, Diet, and Feeding Management

A total of 144 piglets (35 days old; 72 barrows and 72 gilts; Duroc × Large White × Landrace) with 9.45 ± 0.76 kg body weight (BW) were used to perform an 18-day two-way choice test. The piglets were allotted to six dietary treatments with four replicates per treatment and 6 piglets per replicate (including three barrows and three gilts). The four diets used in the experiment included a basal diet containing 52.5% conventional corn (NC, 12.65% gelatinization) and three extruded corn diets containing 12.5% conventional corn and 40% extruded corn with low (LEC; 41.82% gelatinization), medium (MEC; 62.60% gelatinization), and high (HEC; 89.93% gelatinization) degrees of gelatinization. All four diets were tested in pairs to form six treatments: (i) NC vs. LEC, (ii) NC vs. MEC, (iii) NC vs. HEC, (iv) LEC vs. MEC, (v) LEC vs. HEC, and (vi) MEC vs. HEC. The pen used in the experiment had an area of 2.7 × 1.8 m and was equipped with two feeders on each side and an equidistant independent water supply at the opposite wall. The feeders in each pen were switched to the opposite side every three days to avoid any differences associated with the location. Water and feed were provided ad libitum throughout the experimental period. The diets were offered in pellet form, and the composition of the basal diet is presented in Table 1.

#### 2.2.2. Growth Performance and Feed Preference Measurement

Body weight and diet consumption were measured at the end of the experiment to calculate ADFI, ADG, and the feed:gain ratio (F:G). The relative preference for each individual test diet was the ratio of the amount of feed intake of that diet to the total sum of the amount of feed intake of the two diets offered simultaneously in the pair combination.

### 2.3. Experiment 2: Performance Trial

#### 2.3.1. Experimental Animal, Design, Diet, and Feeding Management

A total of 144 piglets (34 days old; 72 barrows and 72 gilts; Duroc × Large White × Landrace) with 9.24 ± 0.55 kg BW were used in a 28-day performance trial. Piglets were weighed and allotted to four dietary treatments and fed with one of the four diets also used in Experiment 1. Each treatment consisted of six replicates, and each replication had 6 piglets (including three barrows and three gilts). The piglets were housed with separate feeders and drinkers. Water and feed were provided ad libitum throughout the experimental period. The diets were offered in pellet form, and the composition of the diets was the same as in Experiment 1, as presented in Table 1. Chromic oxide, which is an indigestible marker, was added to the diets at an inclusion rate of 4 g/kg feed during the last four days of the experimental period.

#### 2.3.2. Growth Performance Measurement

Piglets were weighed on days 0, 14, and 28, and feed consumption was recorded at the same time on a pen basis to calculate ADFI, ADG, and F:G.

#### 2.3.3. Nutrient Digestibility Measurement

Feces and feed were collected from each pen every day during the final four days. The whole feces obtained from each pen were pooled, and H_2_SO_4_ (10%) at 10% (*v*/*w*) was added to part of the feces to determine the crude protein (CP). All feces samples were then dried at 105 °C for 24 h and homogenized before sampling and analysis. The concentrations of nutrients in the feed and feces were analyzed according to the method of AOAC (2007) [11], including ether extract (EE; method 920.39; AOAC Int., 2007), CP (method 920.39; AOAC Int., 2007), DM (method 930.15; AOAC Int., 2007), ash (method 942.05; AOAC Int., 2007), and chromic oxide (method 930.15; AOAC Int., 2007). The organic matter (OM) was calculated as DM minus Ash. The apparent total tract digestibility (ATTD) of CP, EE, DM, and OM was calculated using the following equation:

ATTD% = [(Nutrient/Cr_2_O_3_)diet − (Nutrient/Cr_2_O_3_)feces]/(Nutrient/Cr_2_O_3_)diet × 100% (1)

#### 2.3.4. Serum Physical Indexes and Feeding Hormones Measurement

One barrow and one gilt from each pen were selected for the collection of blood samples from the anterior vena cava on day 14 and at the end of the experiment. The serum samples were centrifuged at 3000× *g* for 10 min at 4 °C and stored at –80 °C until analysis. The concentrations of total protein (TP), albumin (ALB), and total cholesterol (TC) were detected using biuret colorimetry, the bromocresol green method, and the cholesterol oxidase method, respectively. All of the above kits (TP, code no. A045-1-1; ALB, code no. A028-2-1; TC, code no. A111-1-1) were purchased from the Nanjing Jiancheng Bio-Engineering Institute (Nanjing, China). The concentrations of globulin (GLB) were calculated using TP minus ALB. Glucagon-like peptide-1 (GLP-1) and leptin were determined using porcine-specific ELISA kits (GLP-1, code no. H294-1, Nanjing Jiancheng Bio-Engineering Institute, Nanjing, China; leptin, code no. H174, Nanjing Jiancheng Bio-Engineering Institute, Nanjing, China).

#### 2.3.5. 16S rDNA Gene Sequencing Measurement

On day 28, fecal samples were collected via rectal massage from two pigs (one gilt and one ballow) per pen and pooled into one sample for high-throughput sequencing. The total DNA from each fecal sample was extracted using a MicroElute Genomic DNA kit (D3096-01, Omega Inc., Norwalk, CT, USA), according to the manufacturer’s instructions. The V3–V4 fragment of 16S rRNA was amplified using total DNA as a template, and the conserved primers used were 319F (5′-ACTCCTACGGGAGGCAGCAG-3′) and 806R (5′-GGACTACHVGG GTWTCTAAT-3′). Raw data were processed using Greengenes, and sequential analysis, comparison, and annotation were performed using the Ribosomal Database Project (Version 11.3). Illumina MiSeq 2 × 300 bp paired-end was used for sequencing. Operational taxonomic units (OTUs) with a similarity threshold of 97% were determined using R version 3.1.0. Moreover, α- and β-diversity analyses based on the QIIME version 1.7.0-dev were performed to elucidate fecal microflora.

### 2.4. Statistical Analysis

Analyses of BW, ADG, ADFI, and F:G in experiment 1 were performed using one-way ANOVA with a line segment detector (LSD) using SPSS software (version 22.0; SPSS Inc., Chicago, IL, USA). Feed consumption and relative preference in experiment 1 between two diets of a paired combination were analyzed using a *t*-test (SPSS 22.0). Feed consumption, relative preference associated with each individual diet in experiment 1 and growth performance, serum parameters, and nutrient digestibility in experiment 2 were analyzed using linear and quadratic regressions, and then the differences among the treatments were also analyzed using one-way ANOVA with LSD (SPSS 22.0). All statistics and data visualizations for the microbiota were analyzed using R statistical software and related R software or Qiime 1.7.0. Comparisons of alpha diversity and relative abundance of phyla and genera were performed using the Kruskal–Wallis and Wilcoxon rank sum tests. Principal coordinate analysis (PCoA) was performed using weighted UniFrac metrics. Linear discriminant analysis (LDA; >3.0) was performed using the linear discriminant analysis effect size (LEfSe). Data were presented as means and pooled SEM. For BW, ADG, ADFI, F:G, feed preference, ATTD of the nutrients, and fecal microflora, a pen was used as the experiment unit. For plasma parameters, the individual piglet was used as the experiment unit. Differences were considered significant at *p* < 0.05, and a tendency was revealed at 0.05 < *p* < 0.1.

## 3. Results

### 3.1. Growth Performance in Experiment 1

In the two-way choice test, piglets who were given a choice of the degree of gelatinization of the extruded corn showed limited effects on growth performance, including BW, ADG, ADFI, and F:G (Table 2).

### 3.2. Relative Preference and Feed Consumption in Experiment 1

As shown in Table 3, the LEC piglets showed a higher relative preference and feed consumption when they were offered a combination of NC/LEC and LEC/MEC (*p* < 0.05). LEC showed a higher trend in relative preference and feed consumption when piglets were offered a combination of LEC/HEC (0.05 < *p* < 0.01). In the whole feed preference trial, diets supplemented with LEC were the most preferred corn (67.02%) with the highest feed consumption (408.95 g/d). Multiple comparisons showed that LEC markedly increased relative preference and feed consumption compared with that of NC, MEC, and HEC (*p* < 0.05). As the gelatinization degree increased, the relative feed preference and feed consumption quadratically increased (*p* < 0.05).

### 3.3. Growth Performance in Experiment 2

As shown in Table 4, the gelatinization degree markedly affected F:G during the periods of 14–28 days and 0–28 days. Diets supplemented with extruded corn with low or medium gelatinization degrees markedly decreased F:G in the periods of 14–28 days and 0–28 days compared with conventional corn or extruded corn with a high degree of gelatinization (*p* < 0.05). As the gelatinization degree increased, F:G quadratically decreased in the period of 14–28 days (*p* < 0.05). No significant differences were observed in BW, ADG, or ADFI among the four treatments (*p* > 0.05).

### 3.4. Nutrient Digestibility

The nutrient digestibility results are summarized in Table 5. The piglets in the LEC and MEC groups showed a higher ATTD for CP than those in the NC and HEC groups (*p* < 0.05). The ATTD of EE also markedly increased in the MEC group compared with that of the NC and HEC groups (*p* < 0.05). As the gelatinization degree increased, the ATTD of CP and EE quadratically increased (*p* < 0.05).

### 3.5. Serum Physical Indexes and Feeding Hormones

As shown in Table 6, the LEC group showed the highest level of TP and GLB content in the plasma and markedly increased compared with that of the NC group on day 14 (*p* < 0.05). On day 28, no significant difference was observed in the plasma contents of TP, ALB, GLB, and total cholesterol among the four groups (*p* < 0.05). The plasma levels of ORX, GLP-1, and LEP showed no differences on days 14 and 28 among all groups (*p* < 0.05). As the gelatinization degree increased, no linear or quadratic changes were observed in the serum indexes on days 14 and 28.

### 3.6. Fecal Microflora

**Figure 1:**
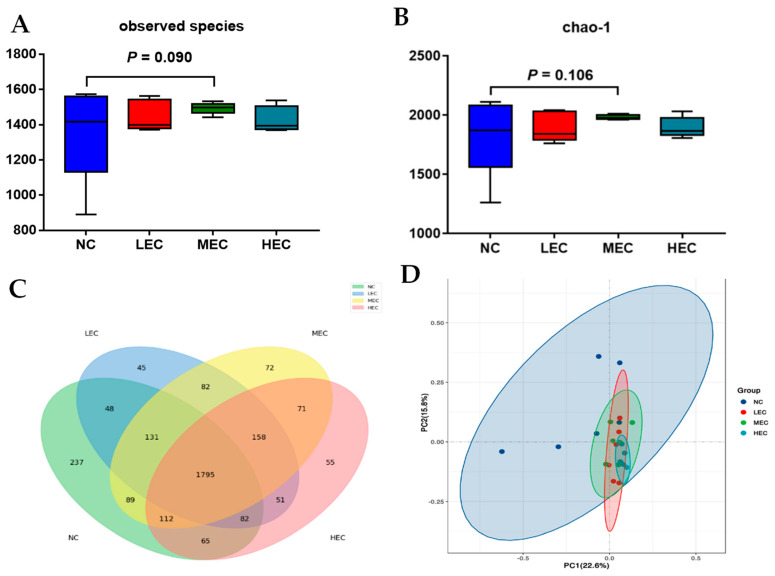
Summary of the microbial community from the fecal samples of piglets in experiment 2 (*n* = 6). (**A**) is the Shannon index reflecting species diversity; (**B**) is the Chao−1 index reflecting species diversity; (**C**) is a Venn diagram summarizing the number of common and unique observed taxonomic units (OTUs) in the microflora; and (**D**) is the PCoA plot based on weighted UniFrac distances.

The effects of the extruded corn on the microbial communities are shown in Figure 1. The MEC group tended to have an increased number of observed species (*p* = 0.090) and Chao-1 index (*p* = 0.106) (Figure 1A,B). Piglets in the four treatment groups shared 1795 OTUs of fecal microflora in the Venn diagram (Figure 1C). NC, LEC, MEC, and HEC contained 237, 45, 72, and 55 OTUs, respectively. The beta-diversity analysis by principal coordinate analysis showed that the microbiota in the NC group were separated from the LEC, MEC, and HEC groups (Figure 1D). The microbiota in the piglets who were fed the extruded corn were highly clustered, regardless of the degree of gelatinization (Figure 1D).

**Figure 2:**
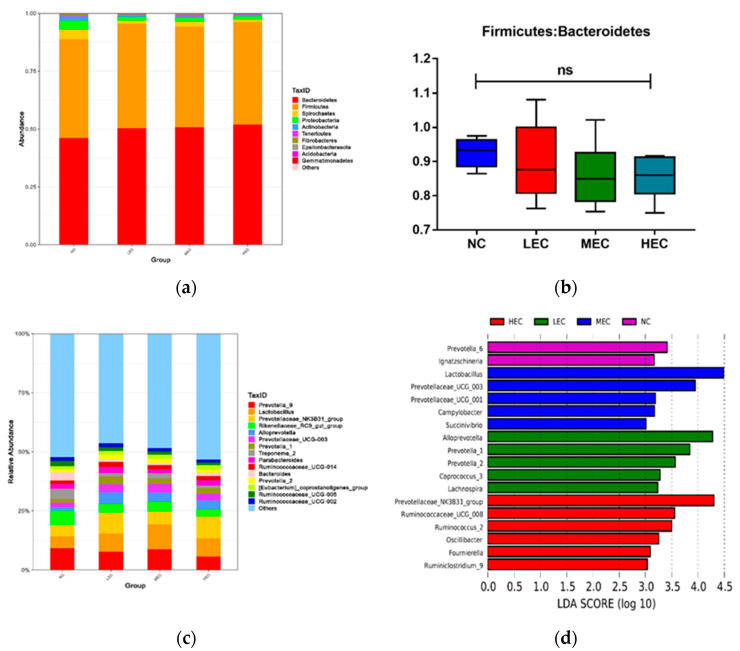
Microbial community structure from the fecal samples of piglets in experiment 2 (*n* = 6). (**a**) represents the relative abundances of the top 15 bacterial strains at the genus level (**b**) is the ratio of Firmicutes/Bacteroidetes. (**c**) is the top 10 relative abundances of bacterial strains at the phylum level. (**d**) represents the LEfSe analysis showing the LDA scores (>3.0). Different superscripts indicate significant differences (*p* < 0.05).

The microbial community structure is presented in Figure 2. At the phylum level, the most abundant phyla were Bacteroidetes and Firmicutes, with an average relative abundance of over 95% of the total bacteria in the piglets. No significant difference was observed in the relative abundance of Firmicutes; however, the relative abundance of Bacteroidetes increased from 46.05% (NC) to 50.37% (LEC), 50.78% (MEC), and 51.92% (HEC) (*p* < 0.05) (Figure 2a). There was no significant difference in the relative abundance ratio of Firmicutes to Bacteroidetes between the groups (Figure 2b). At the genus level, *Prevotella_9* (7.79%), *Lactobacillus* (7.70%), and *Prevotellaceae_NK3B31_group* (7.19%) were the top three genera (Figure 2c). Significant differences in the top 15 genera are shown in Figure 2d. The LEC group had an increased in the relative abundance of *Lactobacillus*, *Prevotellaceae_NK3B31_group*, *Prevotellaceae_UCG-003*, *Allopreotella*, *Prevotella_1*, and *Prevotella_2* compared with the NC group (*p* < 0.05). The MEC group showed a marked increase in *Lactobacillus*, *Allopreotella*, *Prevotellaceae_UCG-003*, and *Prevotella_2* abundance compared with that of the NC group (*p* < 0.05). The HEC group showed an increase in *Prevotellaceae_NK3B31_group*, *Allopreotella*, and *Prevotella_1* abundance compared with that of the NC group (*p* < 0.05). LDA effect size was performed to detect specific bacterial taxa with significantly different abundances among the NC, LEC, MEC, and HEC groups (Figure 2d). Twelve distinct bacterial taxa were identified using a log LDA score threshold of 3.0. *Prevotella 6* and *Ignatzschineria* were found to be enriched in the microbiota of the NC group. *Lactobacillus*, *Prevotellaceae_UCG_003*, *Prevotellaceae_UCG_001*, *Campylobacter*, and *Succinivibrio* spp. were enriched in the MEC group. *Alloprevotella*, *Prevotella1*, *Prevotella_2*, *Coprococcus3*, and *Lachnospira* were enriched in the LEC group. *Prevotellaceae_NK3B31_group*, *Ruminococcaceae_UCG_008*, *Ruminococcus_2*, *Oscillibacter*, *Fournierella*, and *Ruminiclostridium_9* were enriched in the HEC group.

**Figure 3:**
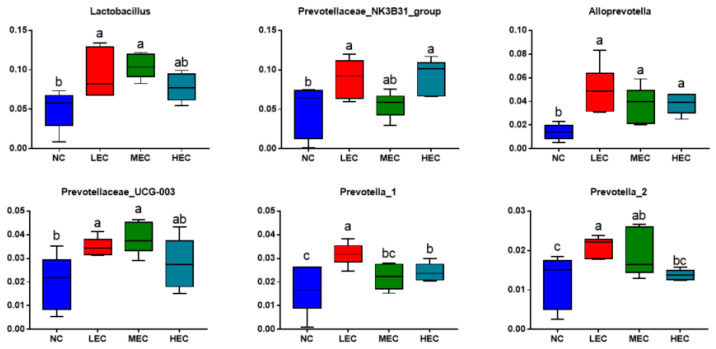
Genus with significant differences in the top 15 from the fecal samples of piglets in experiment 2 (*n* = 6). Different superscripts indicate significant differences (*p* < 0.05). ^a,b^ values differ significantly (*p* < 0.05).

Significant differences in the top 15 genera are shown in Figure 3. The LEC and MEC groups markedly increased their relative abundance of *Lactobacillus*, *Alloprevotella*, *Prevotellaceae_UCG_003*, and *Prevotella_2* compared with the NC group (*p* < 0.05). Meanwhile, the LEC and HEC groups also showed a higher abundance of *Prevotellaceae_NK3B31_group* and *Prevotella_1* than the NC group (*p* < 0.05).

## 4. Discussion

In the two-way choice preference tests, piglets preferred feed containing extruded corn, especially the feed with a 41.82% gelatinization degree. This result was similar to that of Solà-Oriol et al., who noted that extrusion increased pigs’ preferences for several cereals, including corn and naked oats, when the inclusion rate was 30% or 60% [12]. As expected, when the degree of gelatinization of the extruded corn reached 89.93%, the preference decreased to 41%, which was similar to that of conventional corn. This could be a result of the increased feed hardness. Hardness can indicate the maximum force that piglets must compress during chewing. The extrusion process gelatinizes starch and improves its adhesive property, which acts as a binder during pelleting and thus increases the pellet hardness [7,13]. As the degree of gelatinization increased, pellet hardness increased, which decreased feed preference [12]. Starch with a high degree of gelatinization is normally associated with a high water absorption index, which may stick to the mouth, and a greater amount of saliva may be needed to swallow the feed, which affects piglets’ chewing capability [14].

The addition of extruded starch to diets has beneficial effects on nutrient digestibility. Yong et al., reported that supplementary extruded corn increased the in vitro and in vivo apparent digestibility of CP, GE, DM, and starch, both in vitro and in piglets [15]. Liu et al., suggested that the extrusion process increases the ATTD of DM and GE in corn and broken rice in weaning piglets [16]. Several factors can be attributed to increased nutrient digestibility, such as increased enzyme susceptibility, reduced starch resistant content, and decreased starch granules [17,18,19]. In the present study, an increased ATTD of both CP and EE was observed in diets containing extruded corn. We also noted that diets with a medium degree of starch gelatinization (62.60%) resulted in the best digestibility, whereas a high degree of starch gelatinization contributed to low digestibility. These results aligned with Adb et al.’s study, which reported that CP, EE, and DM digestibility in pigeons increased when the starch gelatinization degree decreased from 73.6% to 53.1% [20]. Hongtragul et al., also noted significant quadratic variation between the degree of starch gelatinization and nutritional digestibility [10]. The decreased nutritional digestibility of highly gelatinized corn may be related to intestinal viscosity. Normally, extruded corn with a high degree of gelatinization has high water solubility which decreases the chyme viscosity [18]. The decreased viscosity reflects a short mean transit time, resulting in a reduction in nutrient digestion and subsequent absorption. Over-processing during extrusion may also promote a Maillard reaction between amino acids and glucose which enhances the encapsulation of starch granules via the protein and resistant starch content and thus reduces its digestibility [21,22].

The effects of extruded cereal on the growth performance of piglets varies. Lawlor reported no beneficial effects of extrusion on the growth performance of piglets with different initial body weights [23]. Similar results were reported in Mateos’s study, which used rice and maize diets [24]. Hongtragul’s study showed the positive effects of extruded corn as the piglets’ ADG and ADFI increased [10]. Kotara also found that ADG, ADFI, and G:F increased linearly when the degree of starch gelatinization increased from 23.8% to 81.9% in barley, wheat, and maize diets [9]. One possible explanation for these differences could be the variation in extruded corn quality caused by extrusion conditions, such as steam flow, temperature, and pressure. In the present study, although ADG and ADFI showed no differences among all groups, F:G markedly decreased when the degree of gelatinization of the extruded corn was 41.82% and 62.60%. The nutrient digestibility results were consistent with those of F:G and might be the main reason for the improved feed utilization in the LEC and MEC groups. Meanwhile, we also noted that feed preference had limited effects on ADFI when the piglets were fed a single feed. This difference may be because feed intake is influenced by multiple factors, such as nutritional composition, palatability, and nutritional availability [12]. In the present experiment conditions and diet compositions, nutritional requirements had a stronger effect on feed intake than palatability when piglets were fed the single feed.

The TP is composed of ALB and GLB. ALB is synthesized by the liver and significantly correlated with nutritional status [25]. GLB is synthesized by immune organs and plays an important role in immune responses [26]. In the present study, TP and GLB showed significant differences on day 14, with the highest levels in the LEC group, followed by the MEC group. These results were consistent with the results of F:G and nutrient digestibility, which indicated an increased nutritional status and an increased immunity. GLP-1 and leptin are peripheral appetite regulatory peptides that play important roles in the regulation of feeding behavior. Zhuo et al., noted that extrusion ingredients, including corn and broken rice, elevated the plasma levels of GLP-1 and peptide YY [27]. However, the GLP-1 and leptin levels showed no differences among the four treatments in our present study. These differences may exist because the blood in the experiment was collected after fasting, and the hormone level had dropped to a low point, which made the difference not significant.

The extrusion process elevates nutrient digestibility in the small intestine and alters fermentable substrates in the large intestine, thereby changing the gut microbial community structure. In weaning piglets, the extrusion process of corn or broken rice tends to increase the microbial diversity, as indicated by the increasing Chao-1 and ACE indices [27]. Our present study identified a similar result: extruded corn with a medium degree of gelatinization (62.60%) tended to increase the observed species and Chao-1 index. Generally, higher microbial diversity is associated with higher resilience to environmental challenges and better disease resistance [28]. A study conducted by Moen showed a converse result as cereal extrusion decreased the microbiota diversity in growing pigs [29]. These differences might be due to the growth period or extrusion condition and require further study. Bacteroidetes are Gram-negative bacteria that perform a main role in the maintenance of a healthy state and sophisticated homeostasis safeguarded by microbiota [30]. In the present study, extrusion increased the relative abundance of Bacteroidetes, regardless of the degree of gelatinization. Although Bacteroidetes are normally related to inflammation and obesity-driven dysbiosis [31,32], a study by Vadder et al., indicated that Bacteroidetes strongly correlate with increased levels of short chain fatty acid (SCFA) [33]. The increased abundance of Bacteroidetes can also provide anti-inflammatory effects with certain probiotics [34]. To further identify the effects of increased Bacteroidetes on piglets, we compared the microbial abundance at the genera level among the four groups. One important finding was that *Prevotella*, which is an important genus that belongs to Bacteroidetes, increased in the piglets who were fed extruded corn. A study by Mach et al., reported that the presence of *Prevotella* was associated with increased luminal secretory immunoglobulin A, indicating a positive influence on mucosal immunity [35]. *Prevotella* is also reported to be capable of metabolizing fibers into SCFAs, such as acetate, propionate, and butyrate [36]. Therefore, piglets with a high abundance of *Prevotella* may be better adapted to plant-derived feeds and may confer a performance advantage [37], which in turn may explain the improved feed conversion ratio of extruded corn in the present study. We also noted that extruded corn increased the fecal *Lactobacillus* abundance in piglets, and this effect was positively correlated with the degree of gelatinization. *Lactobacillus* is regarded as a beneficial bacterium that inhibits the growth of some pathogens, indicating healthier gut flora in extruded corn-treated piglets [38]. Wang also found a positive relationship between gelatinized starch and *Lactobacillus* in the sorghum-based diets of growing pigs [39].

## 5. Conclusions

The piglets in this study preferred diets containing extruded corn with a gelatinization degree of 41.82% when they were given a choice. Corn extrusion increased the feed conversion ratio, improved the ATTD of CP and EE, and increased the abundance of Bacteroidetes, *Lactobacillus*, and *Prevotella* in the piglets. Under the experimental conditions, the ideal gelatinization degree of extruded corn for the piglets was approximately 41.82–62.60%.

## Figures and Tables

**Table 1:** Composition and nutrition levels of the basal diets (as-fed basis).

Ingredient	Content	Nutrient Levels ^(2)^	Content
Maize	52.5	Crude protein	18.35 (18.13)
Dehulled soybean meal	16.5	DE (MJ/kg)	14.51
Extrusion full-fat soybean	10	Calcium	0.76 (0.78)
Broken rice	15	Phosphorous	0.62 (0.65)
Fish meal	2	Lysine	1.31 (1.35)
Calcium carbonate	1.1	Methionine + Cystine	0.72 (0.70)
calcium hydrophosphate	0.8	Threonine	0.87 (0.81)
Sodium chloride	0.25		
Coline chloride	0.1		
Lsine hydrochloride 99%	0.42		
DL-methionine 98	0.12		
Threonin 98%	0.18		
Thyptophan 99%	0.03		
Premix ^(1)^	1.00		
Total	100.00		

^(1)^ Premix provided the following per kilogram of basal diet: Cu 125 mg, Zn 70 mg, Fe 100 mg, Mn 40 mg, Se 0.3 mg, I 0.4 mg, vitamin A 7500 IU, vitamin D3 750 IU, vitamin E 25 IU, vitamin K3 2.0 mg, vitamin B1 1.88 mg, vitamin B2 3.75 mg, vitamin B6 2.19 mg, vitamin B12 0.025 mg, nicotinic acid 25 mg, D-pantothenic acid 15.6 mg, folic acid 2.0 mg, and biotin 0.19 mg. ^(2)^ Nutrient levels outside the brackets were calculated values, and the numbers in the brackets were analyzed the levels.

**Table 2:** Growth performance of piglets offered different gelatinization degrees of extruded corn in a two-way choice test in experiment 1 ^1^.

Treatment ^1^	NC/ LEC	NC/ MEC	NC/ HEC	LEC/ MEC	LEC/ HEC	MEC/ HEC	SEM	*p*-Value
Initial BW, kg	9.43	9.46	9.44	9.40	9.46	9.43	0.15	0.403
Final BW, kg	16.04	15.30	15.60	14.77	15.60	15.37	0.29	0.196
ADG, g	366.83	324.33	341.92	298.08	341.33	339.08	9.7	0.221
ADFI, g	633.33	557.87	600.93	537.96	620.14	555.09	19.67	0.439
F:G	1.74	1.73	1.75	1.81	1.81	1.64	0.04	0.848

^1^ NC, basal diet; LEC, basal diet with 40% extruded corn with a low (41.82%) degree of gelatinization; MEC, basal diet with 40% extruded corn with a medium (62.60%) degree of gelatinization; HEC, basal diet with 40% extruded corn with a high (89.93%) degree of gelatinization; ADG, average daily gain; ADFI, average daily feed intake; and F:G, feed gain ratio.

**Table 3:** Feed consumption and relative preferences of piglets offered different gelatinization degrees of extruded corn in experiment 1.

Treatment ^1^	Free Choice a/b	Relative Preference, %				Feed Consumption, g/d			
		a	b	SEM	*p*-Value	a	b	SEM	*p*-Value
1	NC/ LEC	35.22	64.78	6.38	0.004	223.15	410.19	44.11	0.017
2	NC/ MEC	37.44	62.56	7.75	0.106	208.56	349.31	44.25	0.115
3	NC/ HEC	51.14	48.86	8.38	0.904	312.73	288.19	51.31	0.831
5	LEC/ MEC	66.98	33.02	7.00	0.001	360.42	177.55	38.71	0.003
4	LEC/ HEC	69.31	30.69	11.34	0.085	456.25	163.89	85.46	0.083
6	MEC/ HEC	55.01	44.99	7.35	0.537	299.77	255.32	39.33	0.612
Individual diets		Relative preference, %				Feed consumption, g/d			
NC		41.26 ± 8.62 ^b^				248.15 ± 56.40 ^b^			
LEC		67.02 ± 2.26 ^a^				408.95 ± 47.93 ^a^			
MEC		50.02 ± 15.35 ^b^				275.54 ± 88.41 ^b^			
HEC		41.51 ± 9.57 ^b^				235.80 ± 64.41 ^b^			

^1^ NC, basal diet; LEC, basal diet with 40% extruded corn with a low (41.82%) degree of gelatinization; MEC basal diet with 40% extruded corn with a medium (62.60%) degree of gelatinization; and HEC, basal diet with 40% extruded corn with a high (89.93%) degree of gelatinization. ^a,b^ values within rows with different superscript differ significantly (*p* < 0.05).

**Table 4:** Effect of different gelatinization degree of extruded corns on growth performance of piglets in experiment 2.

Treatment ^1^	NC	LEC	MEC	HEC	SEM	*p*-Value		
						Treatment	Linear	Quadratic
BW, kg								
Day 0	9.24	9.23	9.26	9.24	0.12	0.371	0.990	1.000
Day 14	12.05	12.15	12.18	12.20	0.12	0.911	0.674	0.909
Day 28	17.60	18.65	18.09	18.16	0.19	0.313	0.475	0.366
ADG								
0–14 d	200.68	208.45	208.40	211.55	28.54	0.918	0.573	0.826
14–28 d	396.31	464.54	422.76	425.53	54.48	0.291	0.585	0.298
0–28 d	298.49	336.50	315.58	318.54	31.89	0.338	0.450	0.344
ADFI								
0–14 d	434.33	436.35	415.48	426.98	36.74	0.558	0.527	0.758
14–28 d	724.40	771.63	703.57	781.75	88.21	0.434	0.470	0.713
0–28 d	579.37	603.99	559.52	604.37	56.45	0.465	0.726	0.859
F:G								
0–14 d	2.17	2.13	2.03	2.04	0.27	0.397	0.242	0.489
14–28 d	1.84 ^a^	1.67 ^b^	1.66 ^b^	1.85 ^a^	0.18	0.028	0.918	0.039
0–28 d	1.95 ^a^	1.80 ^b^	1.77 ^b^	1.91 ^a^	0.17	0.015	0.653	0.101

^1^ NC, basal diet; LEC, basal diet with 40% extruded corn with a low (41.82%) degree of gelatinization; MEC basal diet with 40% extruded corn with a medium (62.60%) degree of gelatinization; HEC, basal diet with 40% extruded corn with a high (89.93%) degree of gelatinization; ADG, average daily gain; ADFI, average daily feed intake; and F:G, feed gain ratio. ^a,b^ values within rows with different superscript differ significantly (*p* < 0.05).

**Table 5:** The effects of different gelatinization degrees of extruded corn on the ATTD of CP and EE in piglets in experiment 2.

Treatment ^1^	NC	LEC	MEC	HEC	SEM	*p*-Value		
						Treatment	Linear	Quadratic
CP, %	81.44 ^b^	83.47 ^a^	83.45 ^a^	82.39 ^b^	0.23	<0.001	0.157	<0.001
EE, %	83.76 ^b^	84.57 ^ab^	85.64 ^a^	83.46 ^b^	0.26	0.008	0.975	0.013
DM, %	76.90 ^a^	74.04 ^c^	77.50 ^a^	75.80 ^b^	0.31	<0.001	0.831	0.622
OM, %	78.01 ^b^	76.96 ^b^	79.17 ^a^	77.39 ^b^	0.25	0.004	0.980	0.813

^1^ NC, basal diet; LEC, basal diet with 40% extruded corn with a low (41.82%) degree of gelatinization; MEC basal diet with 40% extruded corn with a medium (62.60%) degree of gelatinization; HEC, basal diet with 40% extruded corn with a high (89.93%) degree of gelatinization; CP, crude protein; EE, ether extract; DM, dry matter; and OM, organic matter. ^a,b^ Values within rows with different superscript are differ significantly (*p* < 0.05).

**Table 6:** The effects of different gelatinization degrees of extruded corn on the plasma parameters of piglets in experiment 2.

Treatment ^1^		NC	LEC	MEC	HEC	SEM	*p*-Value		
							Treatment	Linear	Quadratic
TP (g/L)	14d	57.27 ^b^	61.10 ^a^	59.74 ^ab^	58.73 ^ab^	0.51	0.047	0.339	0.558
	28d	56.19	55.99	57.23	57.55	0.38	0.414	0.919	0.752
ALB (g/L)	14d	22.99	24.05	23.85	23.89	0.19	0.201	0.751	0.675
	28d	22.34	22.58	22.65	23.06	0.15	0.495	0.753	0.396
GLB (g/L)	14d	34.28 ^b^	37.05 ^a^	35.89 ^ab^	34.84 ^b^	0.37	0.034	0.200	0.430
	28d	33.85	33.41	34.57	34.49	0.27	0.365	0.991	0.941
TC (mmol/L)	14d	0.95	0.96	0.71	0.80	0.05	0.245	0.353	0.572
	28d	1.07	0.83	0.79	0.98	0.05	0.177	0.930	0.467
GLP-1 (pmol/L)	14d	28.61	29.24	27.65	30.17	0.81	0.773	0.453	0.764
	28d	33.79	34.06	33.22	36.76	1.46	0.582	0.786	0.892
Leptin (ng/mL)	14d	6.99	7.07	6.78	7.38	0.20	0.805	0.155	0.159
	28d	7.97	8.22	8.09	8.47	0.32	0.543	0.180	0.388

^1^ NC, basal diet; LEC, basal diet with 40% extruded corn with a low (41.82%) degree of gelatinization; MEC basal diet with 40% extruded corn with a medium (62.60%) degree of gelatinization; HEC, basal diet with 40% extruded corn with a high (89.93%) degree of gelatinization; TP, total protein; ALB, albumin; GLB, globulin; TC, total cholesterol; and GLP-1, glucagon like peptide-1.^a,b^ values within rows with different superscript differ significantly (*p* < 0.05).

## Data Availability

The data are available on request from the corresponding author.
